# Distinctive Gene Expression Profiles and Biological Responses of Skin Fibroblasts to Nicotinamide Mononucleotide: Implications for Longevity Effects on Skin

**DOI:** 10.3390/biomedicines13102395

**Published:** 2025-09-29

**Authors:** Seongsu Kang, Jiwon Park, Eunbyul Cho, Dohyun Kim, Sanghyun Ye, Eui Taek Jeong, Seung-Hyun Jun, Nae-Gyu Kang

**Affiliations:** R&I Research Division, LG Household and Health Care R&D Center, Seoul 07795, Republic of Korea; franck.kang@lghnh.com (S.K.);

**Keywords:** nicotinamide adenine dinucleotide, nicotinamide mononucleotide, fibroblast, skin aging, longevity

## Abstract

**Background/Objectives:** Enhancement of cellular NAD^+^ mediated by NMN has emerged as a pivotal strategy in modulating the aging process. This study aimed to systematically investigate the anti-aging effects of NMN on human skin fibroblasts, focusing on how the former contributes to the improvement of cellular health and function. This study elucidated the molecular and functional mechanisms by which NMN contributes to the attenuation of skin aging. **Methods:** We performed extensive in vitro and transcriptomic analyses. Human skin fibroblasts were treated with NMN, and the induced biological responses were observed under oxidative stress/photo-aging models. **Results:** Transcriptome analysis revealed distinct gene expression patterns for NAD^+^ and its precursors (NMN, NR, and NAM), showing significant differences between NMN and other precursors (NR and NMN). NMN seemed to be significantly involved in cytokine and chemokine activity. It significantly elevated cellular NAD^+^ levels, activated sirtuin and autophagy pathways, and enhanced mitochondrial function, collectively maintaining cellular homeostasis under stress. Furthermore, it suppressed cellular senescence, promoted cell proliferation, supported extracellular matrix integrity, and accelerated wound healing. **Conclusions:** The study provided essential mechanistic evidence supporting the anti-aging effects of NMN in skin cells and addressed the current lack of scientific validation of NMN-based topical applications. The findings established a solid academic background for future translational research and the development of NMN-based therapeutics and cosmeceuticals.

## 1. Introduction

Nicotinamide adenine dinucleotide (NAD^+^) is a fundamental biomolecule in living organisms and is involved in numerous biological pathways and metabolic processes. It serves predominantly as an essential cofactor for enzymatic activities catalyzed by poly(ADP-ribose) polymerases and sirtuins, thereby exerting a critical regulatory influence on diverse physiological processes [1,2]. Recent research has demonstrated that fluctuations in NAD^+^ levels are “both causes and consequences” of various diseases, such as atherosclerosis, obesity, non-alcoholic fatty liver disease (NAFLD), and neurodegenerative conditions, such as Alzheimer’s and Parkinson’s diseases [2,3,4].

In the last decade, one of the most impressive advances in research on NAD^+^ has been regarding aging. A decline in NAD^+^ is a noticeable hallmark of aging, and its supplementation can ameliorate aging-related metabolic conditions and even extend the lifespan of living organisms [5,6,7,8,9,10]. A decline in NAD^+^ during aging is thought to be a result of an imbalance in NAD^+^ metabolism, driven by three main factors: (1) decreased NAD^+^ biosynthesis due to reduced NAMPT expression [3], (2) enhanced NAD^+^ degradation via the upregulation of the NADase CD38 [11], (3) increased NAD^+^ consumption in response to inflammation and other metabolic stresses [12,13]. NAD^+^ exerts its anti-aging effects by serving as a co-substrate for sirtuins and PARPs, enzymes that regulate chromatin remodeling, DNA repair, and mitochondrial biogenesis [14,15]. Age-related decline in NAD^+^ reduces the activity of these pathways, leading to impaired energy metabolism, accumulation of DNA damage, and cellular senescence. Restoration of NAD^+^ levels reactivates sirtuin-dependent stress resistance and autophagy, enhances genomic stability, and improves stem cell function, ultimately promoting healthy aging [16,17]. However, long-term studies on NAD^+^ have been limited to certain species and organs only, and how it affects cell metabolism during aging is not clearly understood yet [18,19].

Cells utilize three pathways for the generation of NAD^+^, namely salvage, de novo (kynurenine pathway), and Preiss–Handler pathways. Tryptophan and nicotinic acid are converted to nicotinate mononucleotide (NAMN), which is then converted to NAD^+^ through enzymes such as nicotinic acid/nicotinamide mononucleotide adenylyltransferase (NMNAT) and nicotinic acid adenine dinucleotide (NADS), passing through nicotinic acid adenine dinucleotide (NAAD) as an intermediate step. Most NAD^+^ in mammals is generated via the salvage pathway, in which nicotinamide (NAM) is resynthesized to NAD^+^ [20]. Nicotinamide is the most abundant member of the NAD^+^ precursor family, making up an estimated ~80% of the total precursor pool [21]. In the salvage pathway, which is a critical pathway with NAD^+^-consuming and recycling steps, NAM is converted to nicotinamide mononucleotide (NMN) by nicotinamide phosphoribosyl transferase (NAMPT), and NMN is finally converted into NAD^+^ by NMNAT. NMN can also be synthesized from nicotinamide riboside (NR) via NR kinase (NRK)-mediated phosphorylation.

To elevate cellular NAD^+^ levels, NMN has been considered one of the most effective precursors for administration due to the following reasons: (1) higher stability, both biologically and chemically [22,23], (2) smaller molecular size than that of NAD^+^, and (3) easier penetration into cells than NAD^+^ through Slc12a8 [24]. However, the intracellular transportation of both NAD^+^ and NMN has been debated for a long time, due to a lack of clear academic consensus based on limited research [25,26,27].

The effects of NMN on the skin have not been well elucidated to date. Only a few studies have observed that NMN exerts some beneficial effects on the skin or skin cells [28,29], while these studies often combine NMN with other substances and do not focus on topical application. Research on a diverse range of organisms has indicated that NMN is remarkably effective in preventing aging and promoting longevity. However, experimental results on skin cells and the skin itself would be required for the actual utilization of NMN for anti-aging purposes in humans.

This pilot translational study explored the potential of NMN as an intervention for skin aging. We investigated the biological responses of UV-treated skin fibroblasts to NAD^+^ and its precursors using transcriptomic analysis and conducted in-depth research on the effects of NMN on the progression of aging. In transcriptome analysis, each precursor at the same molar concentration exhibited different patterns and potencies for protection against UV-induced aging. In the in vitro experiments to investigate how NMN could ameliorate skin aging and induce longevity effects in diverse aging models (Oxidative stress (acute and chronic stress), and photo-aging model), NMN was found to not only recover the aging-related phenotypes, such as promoting wound healing, cell proliferation, and ECM strengthening (Col type 1 and 3), but also significantly improve and activate the longevity-related cellular mechanisms. Although further in-depth research is needed to understand how NMN exerts its beneficial effects on human skin—particularly regarding skin penetration and metabolic conversion—and how actual gene expression profiles affect cellular phenotypes at the protein level, this study provides foundational evidence supporting the longevity-enhancing effects of NMN on skin physiology, highlighting its potential as a therapeutic intervention for mitigating human skin aging.

## 2. Materials and Methods

### 2.1. Cell Preparation

Fibroblasts (HS68), originally isolated from the foreskin of a white male with aspartoacylase deficiency, were obtained from ATCC (American Type Culture Collection, Manassas, VA, USA). These cells were maintained in DMEM (11965092, Gibco, Waltham, MA, USA) supplemented with 10% fetal bovine serum (FBS; 16000044, Gibco, Waltham, MA, USA) and penicillin–streptomycin (15140122, Gibco, Waltham, MA, USA) at 37 °C in a humidified atmosphere containing 5% CO_2_. To maintain experimental consistency and minimize the influence of cellular aging, only fibroblasts between passages 7 and 10 were utilized. Prior to the start of experiments, cells were detached using trypsin-EDTA (0.25%) (25200072, Thermo Scientific, Waltham, MA, USA) and seeded into 12-well plates (30012, SPL Life Science, Pocheon, Republic of Korea). During treatment with test compounds, the culture medium was switched to DMEM supplemented with 1% FBS to avoid the effects of serum deprivation on NAD^+^ synthesis and autophagy.

NAD^+^, NMN, NR, and NAM were purchased from Aladdin (AL-N196974, AL-N131850, AL-N303138, and AL-N105042, Los Angeles, CA, USA). All test materials were dissolved in sterile distilled water. Untreated control groups received the same amount of water as the vehicle.

### 2.2. H_2_O_2_-Induced Aging Model and UV-Induced Photoaging Model

H_2_O_2_ (300 μM, 4104-4400, Daejung, Seoul, Republic of Korea) was treated to induce oxidative stress and senescence. On the day of the experiment, the existing culture medium was removed, and fresh DMEM containing the test compound along with 300 μM H_2_O_2_ was added to the cells. After incubation for 3 days, the cells were collected for subsequent analyses.

For UV irradiation experiments, the culture medium was first aspirated, and each well was rinsed with Dulbecco’s phosphate-buffered saline (DPBS; Solbio, Seoul, Republic of Korea). Cells were then exposed to ultraviolet light using a UV irradiator (Bio-sun, Vilber Lourmat, Collégien, France), positioned 10 cm above the cell layer. The irradiation parameters included 15 mJ/cm^2^ at 254 nm (narrow-band UVA) and 30 mJ/cm^2^ at 312 nm (narrow-band UVB). Following exposure, DPBS was removed, and DMEM supplemented with the test material was added back to the wells.

### 2.3. Measurement of Cellular NAD^+^ and ATP

Cellular NAD^+^ levels were measured using a commercially available assay kit (PicoSens™ NAD/NADH Assay Kit, BM-NDH-100, Biomax, Guri-si, Republic of Korea) according to the instructions provided by the manufacturer. Briefly, fibroblasts were treated with test material for 24 h. After removing the culture medium, cells were washed three times with PBS and lysed using the provided lysis buffer. Total NAD^+^ and NADH levels were determined enzymatically according to the kit instructions, and NAD^+^ concentrations were calculated by subtracting NADH from total NAD^+^.

Cellular ATP levels were measured using the PicoSens™ ATP Assay Kit (BM-ATP-100, Biomax, Guri, Republic of Korea). Cells were lysed similarly, and ATP quantification was performed via enzymatic reaction as per the manufacturer’s guidelines.

### 2.4. Measurement of Cellular Viability and Wound Scratch Assay

Cell viability of HS68 fibroblasts was assessed using the CCK-8 assay following the manufacturer’s instructions (Cell Counting Kit-8, CK04, Dojindo, Kumamoto, Japan). This assay was also used to determine the relative cell population.

Fibroblasts were plated in 24-well plates and cultured for one day. After removing the culture supernatant, uniform scratches were created using a scratch tool (SPLScar™ Scratcher, 201925, SPL, Pocheon, Republic of Korea). The cells were then rinsed once with PBS and incubated with culture medium containing the test substances. Wound closure was monitored and quantified by microscopy after 24 h.

### 2.5. Cellular Senescence and Oxidative Stress

Cellular senescence was evaluated by detecting β-galactosidase activity, a commonly used marker in prior research. The CellEvent™ Senescence Green Detection Kit (C10850, Thermo Scientific, San Francisco, CA, USA) was employed, and fluorescence intensity was quantified using a microplate spectrophotometer in well-reading mode. Hoechst 33342 nuclear staining dye was used for normalization.

Cellular oxidative stress levels were assessed using the DCFDA assay with a commercially available kit (DCFDA/H2DCFDA Cellular ROS Assay Kit, ab113851, Abcam, Cambridge, UK).

### 2.6. Analysis of Genes, Transcriptome and Protein

Gene expression analysis was conducted using RT-qPCR (Real-time Polymerase Chain Reaction). Total RNA was isolated from fibroblasts with the AccuPrep^®^ Universal RNA Extraction Kit (K-3140, Bioneer, Daejeon, Republic of Korea). Briefly, cells were lysed in the provided lysis buffer containing chaotropic salts to inactivate RNases, and the lysates were applied to a silica-based spin column. After successive washing steps to remove proteins and contaminants, RNA was eluted in RNase-free water. The purity and concentration of isolated RNA were determined spectrophotometrically. A total of 0.5 μg RNA was reverse-transcribed into cDNA using the TOPscript™ cDNA Synthesis Kit (dN6 Mix) (EZ205M, Enzynomics, Daejeon, Republic of Korea) following the manufacturer’s instructions, with reactions carried out in a Veriti 96-Well Thermal Cycler (Applied Biosystems, Waltham, MA, USA). Subsequent cDNA amplification and RT-qPCR were performed using the StepOnePlus™ RT-PCR system (4376600, Applied Biosystems, Waltham, MA, USA) along with the AccuPower^®^ GreenStar™ RT-qPCR Pre-Mix kit (K-6252, Bioneer, Daejeon, Republic of Korea) according to the provided protocols.

RNA sequencing and the following data analysis were performed by Ebiogen Inc. (Seoul, Republic of Korea). The quality of RNA was assessed by determining the RNA Integrity Number equivalent (RINe) using an Agilent 2100 Bioanalyzer (Agilent Technologies, Amstelveen, The Netherlands). Samples with a RINe value above 7.0 were selected for NGS analysis. Libraries were prepared with the QuantSeq 3′ mRNA-Seq Library Prep Kit (Lexogen, Inc., Greenland, NH, USA) following the manufacturer’s instructions. In brief, RNA samples were initially prepared and reverse-transcribed using oligo-dT primers that included Illumina-compatible sequences at their 5′ ends. After enzymatic degradation of the RNA template, second-strand synthesis was carried out with random primers containing Illumina-compatible linker sequences at their 5′ termini. The resulting double-stranded cDNA libraries were purified using magnetic beads to remove leftover reaction components. To finalize adapter incorporation necessary for cluster generation, the libraries underwent PCR amplification followed by an additional purification step to eliminate residual PCR byproducts. High-throughput single-end sequencing of 75 base pairs was performed on the NextSeq 500 platform (Illumina, Inc., San Diego, CA, USA). Differential gene expression and gene ontology analyses were conducted using the Excel-based Differentially Expressed Gene Analysis (ExDEGA 4.0 0) software provided by Ebiogen Inc.

For protein expression analysis via ELISA (Enzyme-Linked Immunosorbent Assay), culture supernatants were collected after 48 h. The Human Pro-Collagen I alpha 1 ELISA kit was obtained from R&D Systems (DY6220-05, Minneapolis, MN, USA). The collagen synthesis rate at each time point was calculated using the following equation:

$$\frac{\left(\mathrm{The}\;\mathrm{amount}\;\mathrm{of}\;\mathrm{collagen}\;\mathrm{synthesized}\right)\mathrm{at}\;t2-\left(\mathrm{The}\;\mathrm{amount}\;\mathrm{of}\;\mathrm{collagen}\;\mathrm{synthesized}\right)\mathrm{at}\;t1}{t2-t1}$$

### 2.7. Evaluation of Sirtuin and Autophagy Activation

Sirtuin activity was evaluated using a modified fluorogenic assay kit specific for SIRT1 (SIRT1 (Sirtuin1) Fluorogenic Assay Kit, 50081, BPS Bioscience, San Diego, CA, USA). Cell lysates were prepared, protein concentrations normalized, and samples incubated with the assay mix. Fluorescence was measured to determine sirtuin activity.

Autophagy was evaluated through autophagosome staining using the Cyto-ID Autophagy Detection Kit (ENZ-51031-0050, Enzo, Farmingdale, NY, USA). Cells were initially washed once with assay buffer supplemented with 5% fetal bovine serum (FBS). The Cyto-ID Green detection reagent and Hoechst 33342 nuclear stain were diluted in assay buffer according to the manufacturer’s protocol, followed by incubation of the cells with the staining solution at 37 °C for 30 min. Post-incubation, cells were washed again with assay buffer containing 5% FBS. Fluorescence intensity was measured using a spectrophotometer at excitation/emission wavelengths of 480/530 nm for Cyto-ID (FITC) and 340/480 nm for Hoechst. The relative autophagosome formation was quantified by normalizing the FITC fluorescence intensity to the Hoechst fluorescence intensity.

For the Western blot analysis of LC3B, total cellular proteins were extracted using M-PER™ Mammalian Protein Extraction Reagent (78501, Thermo Scientific, CA, USA). Protein concentrations were quantified by the bicinchoninic acid (BCA) assay. Proteins were then separated via SDS-PAGE and transferred onto polyvinylidene difluoride (PVDF) membranes. The membranes were blocked with 5% skim milk solution and subsequently rinsed with Tris-buffered saline containing Tween 20 (TBST). Primary antibody incubation was performed overnight at 4 °C using antibodies against LC3B (ab192890, 1:1000, Abcam, Cambridge, UK), p62 (ab91526, 1:1000, Abcam), and β-actin (ab8227, 1:2000, Abcam, 42 kDa). Following three washes with TBST (5 min each), membranes were incubated for 2 h with horseradish peroxidase (HRP)-conjugated goat anti-rabbit IgG secondary antibody (7074S, 1:2000, Cell Signaling Technology, Beverly, MA, USA). After additional washes (three times, 50 min total) with TBST, protein bands were detected via chemiluminescence. For experiments involving autophagy inhibition, bafilomycin A1 was obtained from Aladdin (AL-B101389, Los Angeles, CA, USA).

### 2.8. Mitochondrial Membrane Potential Analysis

As described in previous studies, mitochondrial membrane potential and its dynamics were assessed using tetramethylrhodamine methyl ester (TMRM) [30,31]. Cells were first rinsed with phenol red-free DMEM, then incubated with 25 nM TMRM diluted in phenol red-free DMEM for 30 min. Hoechst dye was added during the final 10 min of this staining period. After staining, cells underwent two washes with phenol red-free DMEM before imaging. For the acute oxidative stress model, cells were treated with 1.5 mM hydrogen peroxide (H_2_O_2_), and TMRM fluorescence was recorded continuously over 60 min using fluorescence microscopy. In the chronic stress condition, cells were exposed to 300 μM H_2_O_2_, with assessments of TMRM fluorescence intensity, cellular morphology, and ATP production (as described in Section 2.3) conducted after three days.

### 2.9. Statistical Analysis

Data are expressed as mean ± standard deviation (SD) from a minimum of three independent replicates. Statistical significance was evaluated using Student’s *t*-test and one-way ANOVA followed by Dunnett’s post hoc test. Differences were considered statistically significant when the *p*-value was below 0.05 (* *p* < 0.05). Comparisons between control and treatment groups were specifically analyzed using Dunnett’s test, with significant differences indicated by # (*p* < 0.05).

## 3. Results

### 3.1. Transcriptome Analysis of NAD^+^ and Its Precursors

**Figure 1:**
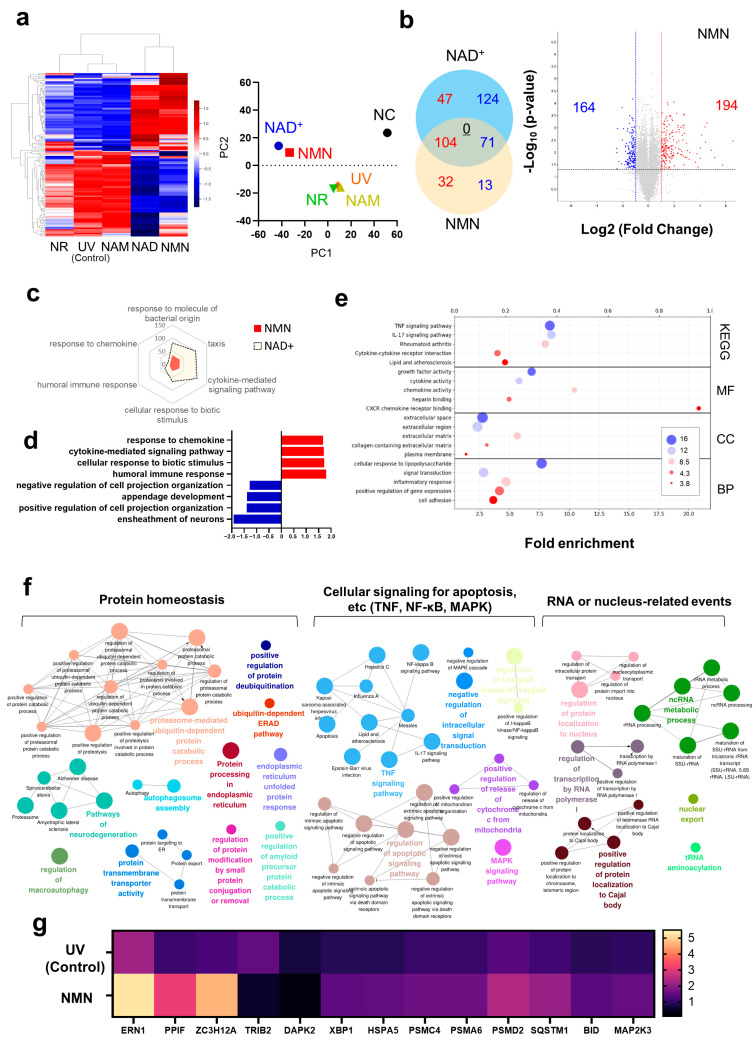
Transcriptome analysis of NMN-treated skin fibroblasts. (**a**) Heatmap analysis and PCA for NAD^+^ and its precursors. Average Z-score was obtained. (**b**) The quantity of genes exhibiting common changes is illustrated using a Venn diagram. Genes with increased expression levels are indicated in red, while those with decreased expression are represented in blue. The number of genes with conflicting expression patterns is underlined. Volcano plot for the DEGs of the NMN-treated group is shown. (**c**) GSEA for NMN- and NAD^+^-treated groups. Commonly occurring gene ontologies are presented. (**d**) Six main gene ontologies for the NMN-treated group are shown. The top and bottom four were selected based on the enrichment scores. (**e**) DAVID analysis for the DEGs from NMN-treated group. (**f**) Network analysis for gene ontologies. Clue-Go was utilized. (**g**) Expression profile for protein homeostasis-related genes. For all analyses, a *p*-value cutoff of 0.05 and a fold-change threshold of 2 were applied. Q-values were calculated by Benjamini–Hochberg (BH) procedure. All experiments were performed in duplicates. 0.1 mM of NAD^+^ and its precursors were treated.

The transcriptomes of UV-irradiated human skin fibroblasts treated with NAD^+^, NMN, NR, or NAM were analyzed. As described in Figure 1a, NAD^+^ and NMN groups showed distinctive gene expression than the control (UV) and other precursors, such as NR and NAM. The gene expression in NAD^+^ and NMN groups was distinct from that in the non-irradiated group (NC). The heat maps, hierarchical clustering, and PCA showed that NAD^+^ and NMN triggered a significant biological response, while the other precursors did not. Interestingly, this observation was in line with other experimental observations that NAM and NR exhibit modest increases in cellular NAD^+^ levels (Supplementary Figure S1). This result indicates that NMN may constitute a more favorable intervention strategy among NAD^+^ precursors.

Hierarchical clustering and PCA showed that NAD^+^ and NMN have similar directions of gene expression with different potencies. When comparing the differential expression genes (DEGs) of the NAD^+^ and NMN-treated groups, both were confirmed to share a total of 175 genes (104 upregulated and 71 downregulated genes). Genes with conflicting expression patterns were not identified. (Underlined number) We compared the common GOs between NAD^+^ and NMN in gene set enrichment analysis (GSEA) (Figure 1d). As expected in DEG or PCA, the gene set size for each common GO term in NAD^+^ was larger than that in the NMN group, implying that NAD^+^ and NMN generate similar biological responses and that NAD^+^ might be more potent than NMN. The top and bottom four GO terms for NMN in terms of Normalized enrichment score (NES) are shown in Figure 1d. They were highly involved in cell–cell signaling. A similar trend was observed in DAVID analysis (Figure 1e). CXCR chemokine receptor binding and cytokine activity in Molecular function, TNF, and IL-17 signaling pathways in KEGG were found to have high fold enrichment scores.

To gain insight into the biological processes influenced by DEGs, we employed ClueGO to construct a network diagram, as shown in Figure 1f. These could be classified into three categories, namely (1) protein homeostasis, (2) cellular signaling for apoptosis, and (3) RNA- or nucleus-related events. Considering that previous studies on aging and NAD^+^ boosting interventions crucially impacted protein homeostasis [32], NMN was considered to exert beneficial effects on the skin in terms of protein homeostasis. Genes related to protein homeostasis were analyzed (Figure 1g), and ERN1, PPIF, and ZC3H12A were found to be greatly increased. Considering that ERN, also well-known as IRE1α, acts as the master key to drive unfolded protein responses (UPR), which also serves as the first checkpoint [33], NMN appears to play a significant role in protein homeostasis, whether in a positive or negative direction. IRE1α acts as a sensor of misfolded or unfolded proteins accumulating in the ER. Upon detecting ER stress, IRE1α oligomerizes and activates its endoribonuclease activity, which splices specific mRNAs such as XBP1 [33]. This leads to increased expression of genes involved in protein folding, quality control, and degradation pathways. Interestingly, our observations are consistent with previous studies reporting that cellular NAD^+^ levels are closely associated with ER stress, and that NAD^+^ replenishment leads to increased ERN1 expression [34]. PPIF plays a role in protein folding by catalyzing the cis-trans isomerization of proline imidic peptide bonds [35] and ZC3H12A regulates mRNA stability, which potentially affects the protein expression levels [36,37]. Regnase-1, also known as MCPIP1, coded by ZC3H12A, is responsible for endonucleolytic cleavage of mRNAs encoding proteins and is involved in various biological functions such as immune homeostasis and cell differentiation or activation [38]. NMN governs protein homeostasis in various ways, such as UPR, protein folding, and mRNA stability. Future studies are essential to clarify whether the observed pathway activation is solely inferred from transcriptomic profiles or experimentally validated. Validation of each key DEG using individual qPCR is also necessary. Such investigations will be critical to confirm the mechanistic insights and strengthen the biological relevance of our findings. It may also contribute to establishing scientific insights in the fields of NAD^+^-related research and aging science.

### 3.2. Biological Response of Human Skin Fibroblasts to NMN

#### 3.2.1. Suppression of Senescence and Activation of Longevity-Related Pathways by NMN

The senescent state is characterized by irreversible cell cycle arrest, expression of a senescence-associated secretory phenotype (SASP), and increased resistance to apoptosis. Senescent cells accumulate in the skin with age, leading to a decline in various aspects of skin function [39]. Although there is a huge gap in understanding how cellular senescence develops age-associated skin phenotypes and changes, numerous studies have demonstrated that senescent fibroblasts and keratinocytes exhibit distorted cellular signaling and homeostasis, which consequently contribute to the development of abnormal functions and morphology at both cellular and tissue levels [40,41].

**Figure 2:**
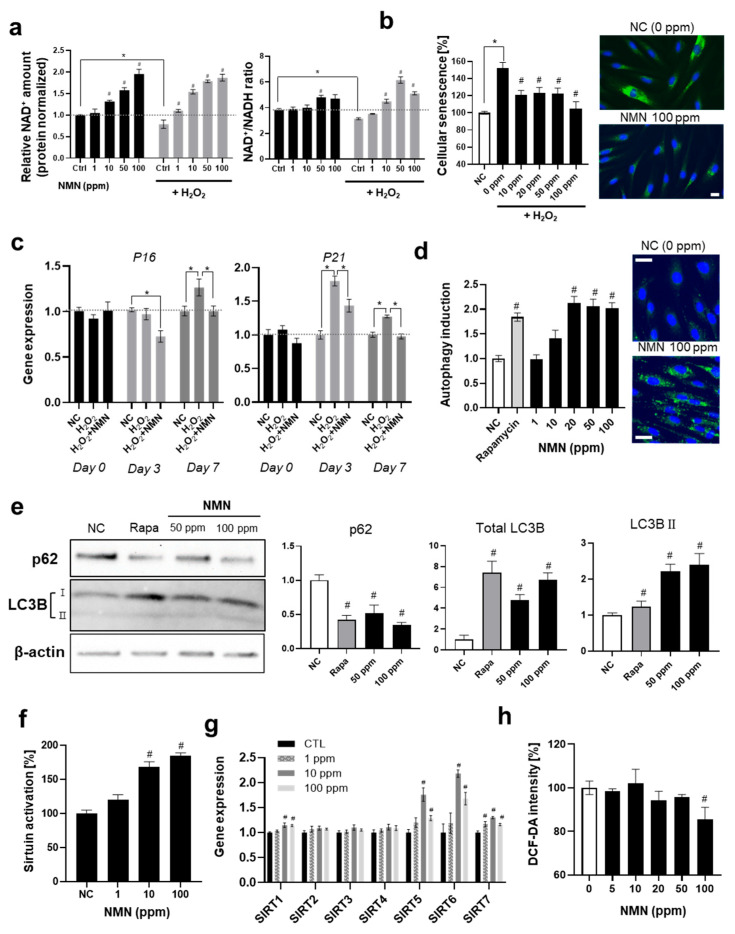
In vitro experiments for the effects of NMN on skin fibroblasts. (**a**) Elevation of cellular NAD^+^ by NMN. Absolute amount of NAD^+^ normalized by protein amount, and NAD^+^/NADH ratio were analyzed. (**b**) Cellular senescence assay. β-galactosidase activity was analyzed with fluorescence microscopy and quantified with a fluorospectrometer. (**c**) Gene expression of aging-related genes, p16 and p21. (**d**) Induction of autophagy by NMN. Autophagic vacuoles were monitored. (**e**) Western blot analysis of autophagy markers in cells exposed to NMN. LC3B/actin and p62/actin ratios were normalized to non-treated group. (**f**) Activation of cellular sirtuins. (**g**) Gene expression of seven sirtuins. (**h**) DCF-DA data analysis for antioxidant effect of NMN on cells. Mean fluorescence of each cell was analyzed. All experiments were performed in triplicate, except for the quantification of NAD^+^ (in quadruplicate). Scale bar, 10 μm. * Significantly different results (Student’s *t*-test, *p* < 0.05). One-way ANOVA (Dunnett’s test) was performed for comparison between control and experimental groups (# Significantly different results (*p* < 0.05)).

In the present study, we investigated the reaction of skin fibroblasts to NMN. NMN effectively increased the cellular NAD^+^ levels at concentrations as low as 1 ppm (Figure 2a). Contrary to the absolute amount of cellular NAD^+^, the NAD^+^/NADH ratio did not increase dramatically, implying that some homeostatic mechanisms are involved in the negative feedback. Interestingly, NMN dramatically restored the H_2_O_2_-induced decrease in cellular NAD^+^ (78.8% compared to NC without H_2_O_2_ treatment) and the NAD^+^/NADH ratio (82.6%), which might have contributed to the anti-senescence effects observed in the subsequent experiments.

Next, we analyzed the anti-senescence effects of NMN on fibroblasts (Figure 2b). In the β-galactosidase activity assay, NMN effectively decreased cellular H_2_O_2_-induced senescence (30.9% decrease at 100 ppm). Gene expression analysis of P16 and P21 showed an anti-senescence effect of NMN (Figure 2c).

Further experiments verified that NMN effectively activated autophagy by approximately 2-fold, which was similar to that by the positive control rapamycin (Figure 2d). In the Western blot analysis evaluating autophagy induction at the protein expression level, both total LC3B and LC3B-II levels increased following NMN treatment, although the increase in LC3B-II was less pronounced than that of total LC3B, while p62 protein levels decreased correspondingly (Figure 2e). These results collectively demonstrate that NMN induces autophagy [42,43]. Cellular sirtuin activation significantly increased in a dose-dependent manner (Figure 2f). This was consistent with previous studies showing that the elevation of cellular NAD^+^ leads to the activation of sirtuins, which triggers autophagy by de-acetylating AMPK, FOXOs, ATGs families and BECN1 [18,44]. Both mechanisms contributed to the modulation of age-related pathways that support cellular survival and function, ultimately ameliorating cellular senescence. Our findings indicate that NMN supplementation elevates intracellular NAD^+^ levels, which in turn activates sirtuins—particularly SIRT1 as described in the previous studies [45]. These NAD^+^-dependent deacetylases play pivotal roles in enhancing mitochondrial function and promoting autophagy, thereby mitigating oxidative stress and DNA damage [46,47,48]. Autophagy, a highly regulated cellular recycling pathway, is indispensable for cellular quality control and the maintenance of homeostasis. Previous studies observed that a deficient autophagy accelerates senescence [49,50] and promotes the development of not only aging-related phenotypes but also diverse pathologic conditions such as Alzheimer’s disease, diabetes and obesity [50,51]. Interestingly, it was observed that autophagy inhibition by bafilomycin A1 compromised the anti-senescence effect of NMN on fibroblasts by approximately 40%, suggesting that the enhancement of autophagy plays a significant role in protecting against cellular senescence (Supplementary Figure S2).

Consequently, this cascade of events contributes to the attenuation of cellular aging and the preservation of tissue homeostasis.

In the gene expression analysis for the seven sirtuins, no significant variation in SIRT2, 3, and 4 was observed, although SIRT1, 5, 6, and 7 increased. NMN significantly elevated SIRT5 and 6 (Figure 2g). Considering that sirtuin5 is primarily localized in the mitochondria and influences mitochondrial functions [52], our observations suggested that NMN exerts beneficial effects on mitochondria, which was consistent with previous studies [18,19,53] and supported by our subsequent experimental results. The antioxidant effect of NMN was observed at concentrations greater than 100 ppm (Figure 2h).

#### 3.2.2. NMN Slows the Progression of Aging and Exerts Protective Effects

**Figure 3:**
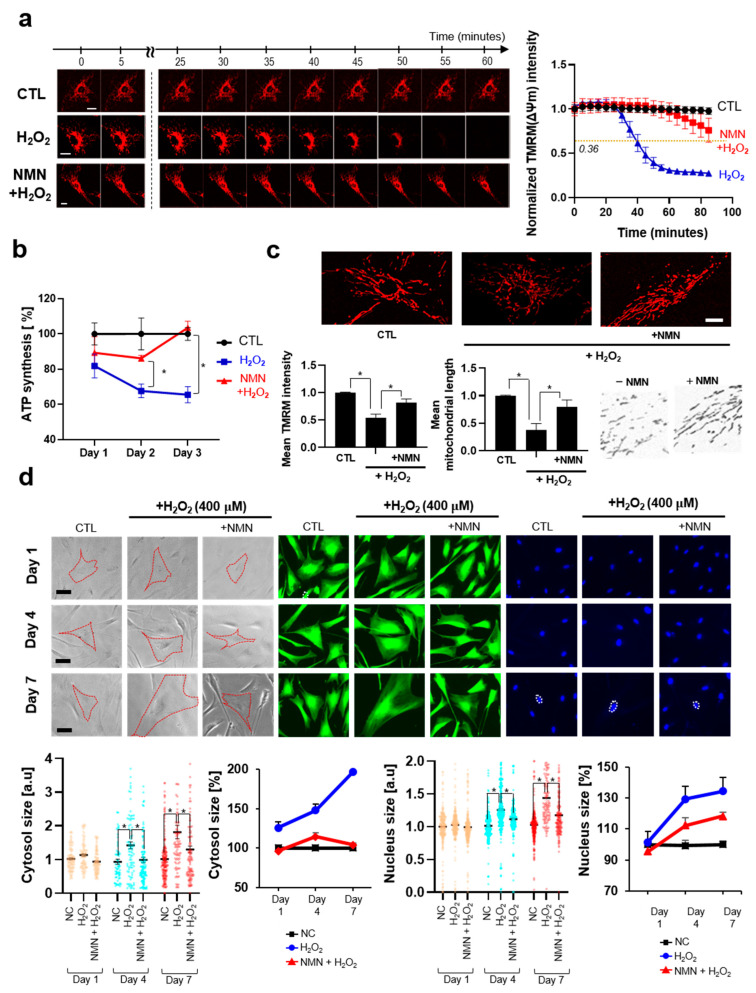
Protective effect of NMN on the progression of aging. (**a**) The decay of fluorescence of TMRM. Fluorescence intensities of fifteen cells were monitored and normalized with respect to the initial point value. Scale bar, 10 μm. (**b**) ATP synthesis of fibroblasts. The number of molecules of ATP was normalized with respect to protein amount. (**c**) Long-term analysis of TMRM intensity in the H_2_O_2_-induced aging model. Mitochondrial morphologies were monitored and analyzed. Scale bar, 10 μm. (**d**) Variations in cell morphologies in the H_2_O_2_-induced aging model. Cytosol size and nucleus size were analyzed using compartment-specific dyes (Calcein AM, and Hoechst 33342). One-hundred cells were analyzed using ImageJ (ver. 1.52). Student’s *t*-test was performed to determine the significance between groups (* Significantly different results (*p* < 0.05)). Scale bar, 20 μm.

In this section, we studied how NMN counteracts the progress of aging over time in the context of cellular hallmarks or features during aging. Numerous studies have pointed out that mitochondrial dysfunction critically contributes to the development of aging and have demonstrated that restoration of mitochondrial functionalities could extend the lifespan in both in vitro and in vivo models [54,55,56]. We assessed the mitochondrial membrane potential (ΔΨm), which refers to the electrical potential difference between the mitochondrial matrix and the intermembrane space, established by proton pumps (complexes I, III, and IV). This parameter is widely recognized as a critical indicator of mitochondrial function [57,58,59]. We verified that NMN effectively protects the H_2_O_2_-induced decrease in ΔΨm in both acute and chronic aging models (Figure 3a–c). In the acute model, the half-life (t1/2), the time required for fluorescence to decay to half its initial intensity, was measured, and NMN was found to significantly increase the t1/2 from 39.8 min to 120.3 min, implying that it slows down the progression of H_2_O_2_-induced aging. A similar trend was observed for ATP production (Figure 3b). In the chronic treatment of H_2_O_2_-induced decrease in TRMM intensity, NMN restored the effect. Improvement of mitochondrial function against oxidative stress appears to be driven by sirtuin activation, both through their enzymatic activity and increased expression, as well as by the subsequent promotion of autophagy and mitophagy. Previous studies have demonstrated that SIRT1 promotes the transcription of genes related to antioxidant defense mechanisms, including superoxide dismutase 2 (SOD2) [60]. Additionally, SIRT3 deacetylates SOD2 to enhance its antioxidant activity and also promotes the expression of SOD2 [46]. It is noteworthy that SIRT5 and SIRT6, members of the SIRT family overexpressed upon NMN treatment, are also highly involved in antioxidant mechanisms through various pathways, such as the SIRT/FOXO3 and NRF2 pathways [46,61]. In addition, overexpression of SIRT5 is estimated to contribute, at least in part, to the protection against mitochondrial fragmentation, as previously reported in the literature [62]. Mitophagy driven by sirtuin activation also seems to contribute to protective effect against oxidative stress, considering that mitophagy can reduce oxidative damage and ROS production in cells [63].

Notably, mitochondrial fission was observed to be promoted by H_2_O_2_ treatment. It is coincident with previous studies that oxidative stress and the progress of aging result in mitochondrial fragmentation [64,65,66]. Fragmented or fission-dominant mitochondria exhibits reduced membrane potential, ATP synthesis, respiration and oxidative phosphorylation and increased production of ROS [64,67]. The predominance of mitochondrial morphology, whether fusion or fission, during cellular senescence and aging is yet to be elucidated owing to discrepancies in experimental results [68,69,70]. Further in-depth and systematic studies of mitochondrial morphology in the context of skin aging would be essential to advance our understanding in this regard. To gain deeper insights into mitochondrial functionality and integrity, subsequent studies could incorporate OCR (Oxygen Consumption Rate) or EACR (Extracellular Acidification Rate) measurements, advanced imaging techniques, and other complementary assays, thereby expanding upon the observations reported here.

We monitored the morphological changes in fibroblasts during aging. During cellular senescence, enlarged cytosol [71] and nuclei [72] have been reported. As mentioned in previous studies, aging-induced fibroblasts exhibit increased cellular size and number of nuclei (Figure 3d). NMN effectively reversed or protected against the morphological changes.

In this study, we employed both acute (1.5 mM, Figure 3a) and chronic (300 μM, Figure 2 and Figure 3b–d) H_2_O_2_ treatment as experimental models to investigate acute cellular stress responses relevant to skin aging. While the acute stress model effectively induces rapid oxidative stress and makes it possible to monitor cellular events or dynamics in real time, it primarily represents acute stress conditions rather than the chronic, cumulative stress that skin experiences during physiological aging. Skin aging in vivo is a gradual process driven by prolonged exposure to low-dose environmental insults, including UV radiation and oxidative stress, which accumulate over time and lead to progressive structural and functional deterioration [73,74]. Therefore, it is important to acknowledge that acute stress models may not fully recapitulate the complex biological mechanisms underlying natural skin aging. Chronic, low-dose stress models, such as repeated sub-cytotoxic UV exposure or extended treatment with mild oxidative agents, could better mimic the slow and sustained damage observed in aged skin. These models allow for the investigation of adaptive cellular responses, long-term alterations in extracellular matrix remodeling, and cumulative DNA damage repair processes that are critical in physiological aging. Future studies incorporating such chronic stress paradigms would provide more comprehensive insights into the molecular and cellular pathways involved in skin aging. In addition, considering the heterogeneity of aging mechanisms across different skin types and environmental contexts, expanding experimental models to include both acute high-dose stress and chronic low-dose stress is essential for a holistic understanding of skin aging biology.

#### 3.2.3. NMN Promotes Cell Proliferation and Restores UV-Induced Decrease in ECM Protein

**Figure 4:**
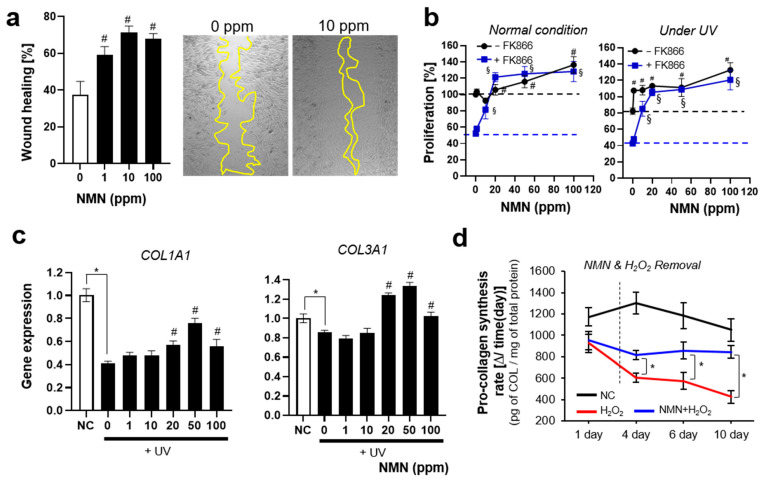
Promotion of cell proliferation and ECM synthesis by NMN treatment. (**a**) Scratch assay. Wound healing ratio was calculated after 24 h of treatment. (**b**) Cell proliferation under various conditions. NAD^+^-depleted condition (FK866 10 μM) is shown in blue. (**c**) Gene expression of COL1A1 and COL3A1. (**d**) Collagen production rate at each time point. The value was calculated as described in Section 2. All experiments were performed in quadruplicate; * significantly different results (Student’s *t*-test, *p* < 0.05); One-way ANOVA (Dunnett’s test) was performed for comparison between control and experimental groups (# Significantly different results (*p* < 0.05)). § compared between FK866 groups (0 ppm vs. test concentrations).

Photoaging induced by UV governs the development of aging-related phenotypes, such as wrinkle formation, hyperpigmentation, and decreased elasticity. UV irradiation significantly decreases the expression of ECM proteins in the skin, such as collagen and elastin, and elevates the expression of protein-degrading enzymes, such as MMPs and TIMPs [75]. UV radiation also induces cellular senescence, and the accumulated senescent cells in the skin are regarded as one of the main causes and consequences of skin aging [40]. Previous studies have shown that a dominant feature of skin aging in both in vitro and in vivo models is delayed cell proliferation and wound healing [76]. We, therefore, investigated how NMN modulates cell proliferation and wound healing. The wound-healing effect of NMN was observed in the cell scratch assay (Figure 4a); even 1 ppm of NMN effectively increased cell migration and proliferation. The maximum effect on wound healing was observed at 10 ppm, whereas the promoting effect seemed to plateau at >10 ppm.

NMN treatment enhanced cell proliferation under both normal and aging conditions (UV irradiation and NAD^+^-depleted condition). Interestingly, its promoting effect was more dominant under UV irradiation and NAD^+^-depleted conditions than under normal conditions, implying that NAD^+^ is required for coping with metabolic stress.

Expression of the two types of collagen was analyzed by measuring the mRNA levels of collagen type 1 (COL1A1) and collagen type 3 (COL3A1). NMN effectively restored the UV-induced decrease in COL1A1 and COL3A1 expression.

Although collagen production by fibroblasts has traditionally been assessed using endpoint analysis, the capacity of fibroblasts to produce type I collagen was continuously monitored throughout the aging process in this study. As expected, skin fibroblasts showed a gradual decrease in synthesis rate of collagen, showing approximately 60% decrease at the final observation. This decrease in collagen production was effectively prevented by NMN treatment. Interestingly, although supplementation with NMN ceased, the protective effect was maintained for over 10 days. The experimental results implied that NMN could exert beneficial effects on ECM production not only on a short time scale but also on a longer time scale through the improvement of cellular components and functions. The experimental results suggested that long-term application of NMN to the skin could provide the best results. Taken together, these findings highlight the high potential of NMN as an effective agent for protecting against and delaying skin aging, supporting its further development as a promising intervention in skin anti-aging strategies.

## 4. Conclusions

In this study, we conducted a systematic investigation into the deceleration of cellular aging induced by NMN in human skin fibroblasts through integrative transcriptomic profiling and functional assays.

Transcriptomic analysis revealed that NAD^+^ and NMN induce robust and unique gene expression profiles, distinct from those elicited by NR and NAM. Among those, NMN notably promoted cellular responses associated with protein homeostasis, RNA regulation, and anti-apoptotic signaling, all of which are hallmarks of cellular longevity.

Our experiments showed that NMN elevates cellular NAD^+^ levels, activates sirtuin and autophagy pathways, and improves mitochondrial function—all of which contribute to maintaining cellular homeostasis under oxidative and chronological stress, as described in previous studies involving other cell lines. Notably, NMN suppressed cellular senescence, enhanced proliferation, supported extracellular matrix integrity, and promoted wound healing, thereby counteracting skin aging across both molecular and phenotypic dimensions.

The strength of this study lies in its multi-angle approach to evaluate how NMN slows down aging at the cellular level, rather than merely reversing age-related phenotypes. By demonstrating that NMN not only mitigates existing damage but also actively decelerates the progression of aging, we highlighted its potential as a long-term skin longevity modulator.

The findings provided a strong foundation for future translational research and supported the development of NMN-based therapeutic or cosmetic interventions aimed at preserving youthful skin and delaying the onset of age-related decline.

This study has several limitations in the context of translational relevance. First, the experiments were conducted in vitro using human neonatal foreskin fibroblasts under artificial aging models. Second, 3D culture and ex vivo experiments were not performed. Based on the findings of this study, further in-depth investigations on ex vivo and in vivo are warranted. Such studies, particularly those investigating how NMN induces biological responses and is effectively delivered and metabolized in skin tissue, will provide more rigorous validation of NMN’s anti-aging effects on the skin.

Despite the growing market for NMN-based supplements and skincare products, scientific evidence supporting their efficacy, especially in skincare, remains limited. To date, the effects of NMN on the skin remain incompletely understood. Although a limited number of studies have reported beneficial effects of nicotinamide mononucleotide (NMN) on skin or skin cells, these investigations frequently involve co-administration with other compounds and rarely address the diverse biological responses of skin cells to NMN or its potential for topical application. The current study could provide foundational mechanism-based evidence of the anti-aging effects of NMN in skin cells, thereby offering a critical scientific basis for its application in consumer products. Our findings not only validated the biological potential of NMN but could also serve as a pivotal reference point for the rational development of NMN-containing cosmeceuticals and therapeutics. Looking ahead, NMN is considered to have considerable promise not only in anti-aging skin care but also in broader therapeutic strategies aimed at healthy aging. Its significance lies in enabling innovative interventions that may contribute substantially to improving quality of life, supported by further in-depth translational studies.

## Data Availability

The Data supporting the findings of this study are available from the corresponding author upon request.

## Supplementary Materials

The following supporting information can be downloaded from https://www.mdpi.com/article/10.3390/biomedicines13102395/s1: Figure S1 Quantification of cellular NAD^+^ when NAD^+^ and its precursors (NAM, NMN, NR) were treated; Figure S2 Cellular senescence assay when autophagy inhibitor was treated; Table S1 GSEA analysis for NAD^+^, and NMN treated groups.
