# Supplementary material for: Distinctive Gene Expression Profiles and Biological Responses of Skin Fibroblasts to Nicotinamide Mononucleotide: Implications for Longevity Effects on Skin
Source: Biomedicines. 2025 Sep 29;13(10):2395. doi: 10.3390/biomedicines13102395 (PMC12561839; doi:10.3390/biomedicines13102395)
Supplement: Supplementary file 1 [file biomedicines-13-02395-s001.zip › biomedicines-3830489-supplementary.pdf]

biomedicines

MDPI

Communication

Distinctive Gene Expression Profiles and Biological Responses of Skin Fibroblasts to Nicotinamide Mononucleotide: Implications for Longevity Effects on Skin

Seongsu Kang, Jiwon Park, Eunbyul Cho, Dohyun Kim, Sanghyun Ye, Eui Taek Jeong,Seung-Hyun Jun * and Nae-Gyu Kang *

R&I Research Division, LG Household and Health Care R&D Center, Seoul 07795, Republic of Korea; franck.kang@lghnh.com (S.K.)

* Correspondence: Correspondence: junsh@lghnh.com (S.-H.J.); ngkang@lghnh.com (N.-G.K.)

NAD⁺

Gene Set	Description	Size	Leading Edge Number	ES	NES	P Value	FDR
GO:0002237	response to molecule of bacterial origin	82	36	0.83974	3.7775	<2.2e-16	<2.2e-16
GO:0071216	cellular response to biotic stimulus	66	23	0.84138	3.6992	<2.2e-16	<2.2e-16
GO:0019221	cytokine-mediated signaling pathway	108	25	0.76672	3.5947	<2.2e-16	<2.2e-16
GO:0050900	leukocyte migration	78	17	0.75995	3.4404	<2.2e-16	<2.2e-16
GO:0042330	taxis	102	33	0.71916	3.2999	<2.2e-16	0.00017149
GO:0006959	humoral immune response	33	11	0.8817	3.4317	<2.2e-16	0.00020579
GO:0070555	response to interleukin-1	32	12	0.84387	3.285	<2.2e-16	0.00044098
GO:0002274	myeloid leukocyte activation	46	13	0.76992	3.2458	<2.2e-16	0.0011576
GO:1901342	regulation of vasculature development	73	22	0.72549	3.2016	<2.2e-16	0.0025152
GO:1990868	response to chemokine	19	9	0.91309	3.1584	<2.2e-16	0.00391
GO:0007274	neuromuscular synaptic transmission	5	5	-0.70721	-1.9816	<2.2e-16	0.1219
GO:0009187	cyclic nucleotide metabolic process	9	9	-0.49736	-1.8992	0.0088889	0.14234
GO:0006907	pinocytosis	6	6	-0.59865	-1.8088	0.017857	0.17805
GO:0043543	protein acylation	21	17	-0.2973	-1.7064	0.055556	0.17837
GO:1990806	ligand-gated ion channel signaling pathway	5	5	-0.75528	-2.0169	<2.2e-16	0.19061
GO:0048193	Golgi vesicle transport	80	80	-0.14984	-1.7138	<2.2e-16	0.19985
GO:0071826	protein-RNA complex organization	55	46	-0.1959	-1.7469	<2.2e-16	0.20184
GO:0031023	microtubule organizing center organization	23	22	-0.26588	-1.6494	0.046512	0.21065
GO:0043113	receptor clustering	13	13	-0.33813	-1.5034	0.03871	0.28339
GO:0016482	cytosolic transport	31	31	-0.23597	-1.5609		

NMN

Gene Set	Description	Size	Leading Edge Number	ES	NES	P Value	FDR
GO:0006959	humoral immune response	12	9	0.95916	1.8041	<2.2e-16	<2.2e-16
GO:0007272	ensheathment of neurons	8	8	-0.70145	-1.926	<2.2e-16	0.0072598
GO:0071216	cellular response to biotic stimulus	26	13	0.8783	1.7283	<2.2e-16	0.044091
GO:0019221	cytokine-mediated signaling pathway	31	20	0.86058	1.7124	<2.2e-16	0.056501
GO:0008277	regulation of G protein-coupled receptor signaling pathway	7	2	0.93079	1.6778	0.0021529	0.13227
GO:1990868	response to chemokine	10	7	0.90964	1.6811	0.0020597	0.14599
GO:0006898	receptor-mediated endocytosis	9	2	0.89064	1.6364	0.012333	0.2917
GO:0019932	second-messenger-mediated signaling	16	1	0.85579	1.641	0.0050454	0.30635
GO:0002237	response to molecule of bacterial origin	35	16	0.81584	1.6206	0.001	0.36338
GO:0042330	taxis	33	8	0.81215	1.6141	<2.2e-16	0.36394
GO:0031331	positive regulation of cellular catabolic process	6	2	0.83987	1.4692	0.053393	0.37295
GO:0048736	appendage development	9	9	-0.50581	-1.3869	0.076923	0.39542
GO:0031346	positive regulation of cell projection organization	7	7	-0.53757	-1.398	0.078125	0.54739
GO:0031345	negative regulation of cell projection organization	6	6	-0.51873	-1.2782	0.092784	0.58223
GO:0010975	regulation of neuron projection development	12	12	-0.3695	-1.1379	0.33333	0.71934

GO:0090596	sensory organ morphogenesis	12	12	-0.3695	-1.0263	0.52941	0.72598
GO:0010927	cellular component assembly involved in morphogenesis	8	8	-0.37101	-1.0067	0.46296	0.72933
GO:0031032	actomyosin structure organization	8	8	-0.37101	-0.9873	0.4375	0.73614
GO:0061383	trabecula morphogenesis	5	5	-0.5	-1.0987	0.27273	0.76645
GO:0050803	regulation of synapse structure or activity	5	5	-0.42529	-0.93679	0.52941	0.78342

Supplementary table S1. GSEA analysis for NAD^+, and NMN treated groups. For all analyses, a p-value cutoff of 0.05 and a fold-change threshold of 2 were applied.

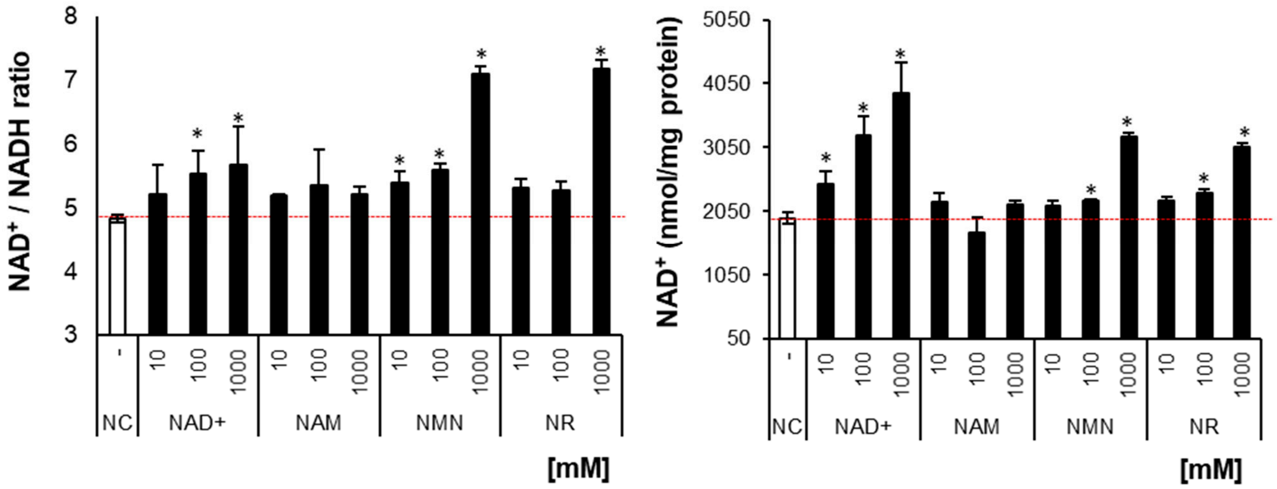

Supplementary Figure S1. Quantification of cellular NAD+ when NAD+ and its precursors (NAM, NMN, NR) were treated. All experiments were performed in triplicate. * Significantly different results ($p < 0.05$)

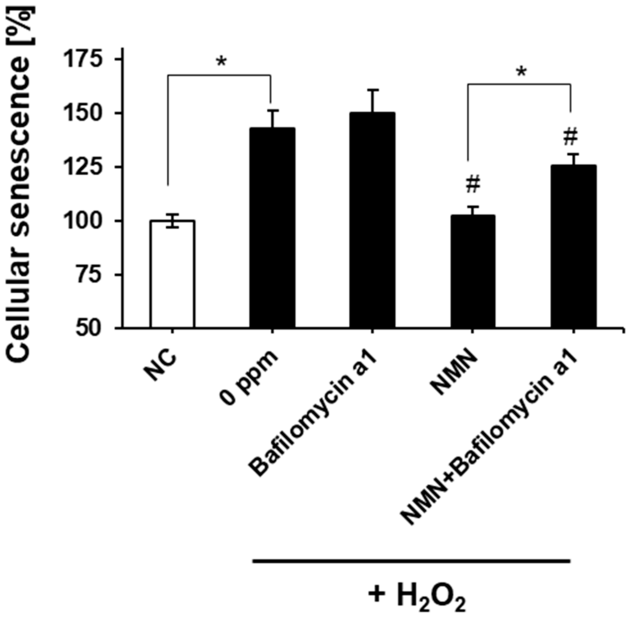

Supplementary Figure S2. Cellular senescence assay. β-galactosidase activity was analyzed with fluorescence microscopy and quantified with a fluorospectrometer. All experiments were performed in triplicate. 100 ppm of NMN and 50 nM of bailomycin a1 were treated. *Significantly different results (Student's t-test, $p < 0.05$). One-way ANOVA (Dunnett's test) was performed for comparison between control and experimental groups (# Significantly different results ($p < 0.05$)).
